# Comparative pulmonary functional recovery after Nuss and Ravitch procedures for pectus excavatum repair: a meta-analysis

**DOI:** 10.1186/1749-8090-7-101

**Published:** 2012-09-29

**Authors:** Zhenguang Chen, Ela Bella Amos, Honghe Luo, Chunhua Su, Beilong Zhong, Jianyong Zou, Yiyan Lei

**Affiliations:** 1 Department of Thoracic Surgery, the First Affiliated Hospital, Sun Yat-sen University, Guangzhou, People's Republic of China; 2 Lung Cancer Research Center, Sun Yat-sen University, Guangzhou, People's Republic of China; 3 Department of Thoracic Surgery, Department of Cardiothoracic Surgery in Huangpu Division, the First Affiliated Hospital, Sun Yat-sen University, Guangzhou, People's Republic of China; 4 Department of Thoracic Surgery, Cancer Center, Sun Yat-sen University, Guangzhou, Guangdong Province, People's Republic of China; 5 Department of Thoracic Surgery, the Fifth Affiliated Hospital, Sun Yat-sen University, Zhuhai, Guangdong Province, People's Republic of China

**Keywords:** Thoracoscopy, Pectus excavatum, Pulmonary function

## Abstract

**Background:**

Pectus excavatum (PE) is a common chest wall malformation, with surgery being the only method known to correct the defect. Although the Nuss and Ravitch procedures are commonly used, there is no consensus as to whether surgical repair improves pulmonary function. We therefore investigated whether pulmonary function recovers after surgical repair, and if recovery is dependent on the type of procedure or time after surgery.

**Methods:**

Literature searches were performed using PubMed, EMBASE, Health Periodicals Database, and CNKI (Chinese National Knowledge Index) from January 1990 to December 2007. The following keywords were used: pectus excavatum, chest wall deformity, funnel chest, pulmonary function, respiratory, lung function, and pectus severity index. The primary outcome of interest was possible changes in pulmonary function following surgical repair.

**Results:**

Meta-analysis of 23 studies showed that, although there was evidence of statistically significant heterogeneity among studies (Chi-square, 17.11, *p* < 0.05), changes in pulmonary functional indices, including forced expiratory volume over 1 s (FEV1), forced vital capacity (FVC), vital capacity (VC), and total lung capacity (TLC), were similar 1 year after the Ravitch and Nuss procedures. Several years after surgery and bar removal, however, the changes in pulmonary functional indices significantly favored the Nuss procedure.

**Conclusions:**

Pulmonary function tends to improve after the surgical correction of pectus excavatum. Although the Nuss procedure was not significantly better 1 year after surgery, long-term postoperative pulmonary function improvement was significantly better after bar removal.

## Background

Pectus excavatum (PE) is a common congenital deformity of the anterior chest wall in children and adolescents [1]. Although it is not life-threatening and does not manifest in severe functional pathophysiology of the thoracic organs, the chest appears concave and the heart is commonly displaced to the left midaxillary line slightly below the axilla. The most common configuration in PE is a symmetric depression involving the lower three quarters of the sternum, extending laterally almost to the costochondral junctions [2,3]. The depth and extent of depression determine the degree of cardiac compression, and the posterior displacement of the lower sternal segment may move the heart and middle mediastinal contents posteriorly and to the left, creating a rotational effect in patients with severe PE [4]. This can cause cardiopulmonary dysfunction due to elevated right atrial pressure and impaired diastolic filling of the right atrium and ventricle, resulting in decreased stroke volume and cardiac output during upright exercise, a defect regarded more as a disorder of compliance of the right side of the heart than the output of the ventricle [5]. Decreased right ventricle filling due to impaired function of the right atrium is also responsible for reduced stroke during exercise [6].

The more important aspect of the pathophysiology of PE is that the depth and extent of the sternal depression determine the degree of pulmonary compression, which in turn determines the degree of incapacitation, often causing impairment of pulmonary function [7,8]. Although this impairment, which manifests as reduced total lung capacity and inspiratory vital capacity, is often observed in patients with PE, some of these patients show normal respiratory function [9]. In contrast to current understanding of cardiac pathophysiology in PE, it is unclear whether pulmonary function recovers after the surgical correction of PE.

The standard surgical treatment of PE since 1949 has been the Ravitch technique. This method, however, was modified to meet requirements related to cosmetics, orthopedics, and effectiveness in relieving symptoms [10]. The modified Ravitch procedure requires exposure of the sternum and surrounding area, removal of abnormal cartilage, and fixation of the sternum in a more normal position with a metal bar, which remains in place for at least 1 year and is later removed surgically [11]. The more recently developed Nuss procedure relies on internal bracing, which is made possible by the flexibility and malleability of the costal cartilages, and does not include a large incision to resect abnormal cartilage [12,13]. Following the Nuss procedure, the steel strut must remain in place for approximately 2–4 years to properly reform the chest. The rationale for this technique was based on the malleability of the chest, chest reconfiguration, and bracing of the bar. The anterior chest wall has been regarded as more malleable than other skeletal structures, making the Nuss procedure seemingly ideal for the correction of PE [14,15]. However, despite many studies, there is as yet no consensus as to whether surgical repair significantly improves pulmonary function. There is a need to assess whether and to what extent pulmonary function, as assessed by severity index, is impaired in PE patients, and to investigate actual changes in pulmonary function caused by surgical correction. We therefore performed a meta-analysis to assess whether pulmonary functional recovery is dependent on the type of surgical correction or to time after the operation.

## Methods

### Study identification and selection

Eligible studies were identified by searching the PubMed, EMBASE, Health Periodicals Database, and CNKI (Chinese National Knowledge Index) databases for relevant reports published from January 1990 to December 2007. The following keywords were searched, either alone or in various combinations: pectus excavatum, chest wall deformity, funnel chest, pulmonary function, respiratory, lung function, age group, follow up, costal cartilage, and pectus severity index. The titles and abstracts of studies obtained were examined to exclude any articles that were clearly irrelevant. The full texts of the remaining articles were retrieved, and each paper was read to determine whether it contained information on the topic of interest.

Inclusion criteria for this meta-analysis included the following: (1) reports that included quantitative measurements of preoperative and postoperative pulmonary function; (2) reports published in the English or Chinese language; (3) reports indexed between January 1990 and December 2007. Abstracts from conference proceedings, doctoral dissertations, and masters' theses were not included because those sources are unlikely to report substantive research findings that are not published elsewhere. Studies meeting the inclusion criteria were examined to ensure that the same subjects were not included in more than one study [16].

### Data extraction

The following information was extracted from each study: study characteristics, physical characteristics of subjects, type of surgical repair performed, pulmonary outcomes, major presenting symptoms, and postoperative complications. Because not all studies reported the same index of pulmonary function, we elected to place the indices reported in each study into pulmonary function global categories. When specific results were not reported, we used available tabular data to calculate them. The indices included in this meta-analysis (Table 1) represent components that individually or collectively determine pulmonary function, including forced expiratory volume over 1 second (FEV1), forced vital capacity (FVC), vital capacity (VC), and total lung capacity (TLC).

**Table 1:** **Pulmonary function, surgical repair,patient satisfaction and follow-upin the studies usedin our meta-analysis**

**Author**	**Pulmonary indices**	**Surgical technique**	**Follow-up**
Aronso [17]	VC, FEV1, FRC, TLC, MEF50	Nuss	6 m
Bawazir [18]	VC, FEV1, FVC, TLC	Nuss	2 yrs
Borowitz [19]	FVC, FEV1, TLC, FEF25-75 RV/TLC	Nuss	1 yr
Croituru [20]	NR	Nuss	NR
Davis [21]	NR	Ravitch	NR
Fonkalsrud [22]	NR	Ravitch	12.6 yrs
Kaguraoka [23]	VC, FEV1, MVV, TLC	Sternocostal elevation	6 m
Hu [24]	VC, FEV1, V25, FVC, TLC	Ravitch	4.2 yrs
Jiang [25]	FEV1, FVC, MVV, MMEF V75-50-25	Ravitch	NR
Kowalewski [26]	FEV1, MVV, IVC	Ravitch	1 yr
Lawson [27]	FVC, FEV1, FEV25-75	Nuss	2 yrs
Quigley [28]	FVC, RV, RV/TLC	Ravitch	8.5 m
Morshuis [29]	IVC, FEV1, FRC, RV, TLC	Daniel	8.1 yrs
Sigalet [30]	VC, FEV1, FVC, TLC	Nuss	2 yrs

Note: IVC: inspiratory vital capacity; FEV1: forced expiratory volume measured over one second; FRC: functional residual capacity; FVC: forced vital capacity; m: month; MVV: maximum voluntary ventilation; NR: not required; RV: residual volume; TLC: total lung capacity; V25: maximal expiratory flow in 25% vital capacity; VC: vital capacity; yrs: years.

### Statistical analysis

The primary outcome of interest was change in pulmonary function following surgical repair. Statistical analysis was performed preferentially using the Cochrane Review Manager Software (version 4.2.10). The Statistical Package for the Social Sciences software (version 13.0; SPSS Inc., Chicago, IL, USA) and Microsoft Office Excel 2007 were also used. Results were considered statistically significant if *p* < 0.05. In addition, 95% confidence intervals were reported.

## Results

### Study characteristics

The initial computerized searches identified 171 potentially relevant articles using the search terms ‘pectus excavatum’ and ‘pulmonary’. We also reviewed reference lists from original and review articles to identify any studies not previously identified from the computerized searches. Overall, we identified 23 studies for our analysis (Table 2).

**Table 2:** **Characteristics of the studiesused in our meta-analysis**

	**Author**	**Cases**	**Race**	**Gender**		**Age**		**Mean age**	**Mean PSI**
				**M**	**F**	**> 14 yr**	**≤ 14 yr**		
Ravitch	Pan [31]	268	Asian	213	55	0	268	4.5	
	Jo [32]	16	Asian	14	2			8.8	4.0
	Jiang [25]	27	Asian	24	3	0	27	5.0	
	Hu [24]	137	Asian	113	24	0	137	4.8	
	Molik [9]	68	Western			0	68	12.6	
	Kowalewski [26]	34	Western	24	10			13.4	
	Fonkalsrud [22]	139	Western	111	28			17.3	4.9
	Davis [21]	69	Western	58	11	22	47	14.5	
	Boehm [33]	7	Western	5	2	0	7	17.8	
	Total	769		566	135	22	557	9.1	
Nuss	Fan [20]	87	Asian	62	25	7	80	5.8	
	Jo [32]	107	Asian	79	28			7.9	4.3
	Kim [34]	51	Asian	40	11	12	39	13.2	4.74
	Uemura [35]	107	Asian	75	32	101	6	7.5	6.1
	Watanabe [36]	53	Asian	38	15	6	47	9.0	4.96
	Croitoru [20]	303	Western	243	60			12.4	
	Dzielicki [37]	461	Western	362	99	229	232	15.2	
	Aronso [17]	35	Western	28	7	35	0	23.0	
	Aronso [17]	141	Western	105	36	0	141	13.0	
	Fonkalsrud [22]	68	Western	49	19			12.0	4.2
	Bawazir [18]	48	Western					13.5	3.9
	Molik [9]	35	Western			0	35	9.5	
	Sigalet [30]	26	Western			0	26	13.2	4.5
	Boehm [33]	21	Western	20	1	0	21	14.4	
	Borowitz [19]	10	Western	10	0			13.4	
	Total	1555		1113	333	390	629	12.4	
Overall		2476		1796	503	491	1259	11.6	

Studies that seemed to include overlapping samples were excluded, with only one of each used. Therefore, we used the study by Hu et al. because it provided the necessary statistical information [24]. Since the studies by Quigley et al. and Haller et al. appeared to use the same sample of pectus excavatum patients [4,27], we combined the results of these two studies and analyzed them as one study. Kowalewski et al. published two studies in 1998 and 1999 using samples that overlapped; thus, only the first study, which included groups of patients with both moderate and severe pectus excavatum, was included in the meta-analysis [26]. Our meta-analysis of pulmonary function included 15 studies (Table 1) performed in the United States, Canada, Japan, Poland, Netherlands, and China. Pectus severity index was not reported in all these studies used. Techniques for estimating pectus severity index included chest radiography (CXR), chest computed tomography (CT), and calipers. We could not assess the relationship between pectus severity and average ES because the included studies did not report these results. Five of the 23 studies included patients who underwent both the Ravitch and Nuss procedures, 6 included patients who underwent the Ravitch repair surgical procedure, 11 included patients treated by the Nuss procedure, 1 study used sternal eversion, and 1 used the Daniel procedure.

The meta-analysis included a total of 2476 patients with PE, including 921 who underwent open surgical repair (Ravitch + Daniel) and 1555 who underwent the minimally invasive Nuss technique. Average patient age was 11.6 years, with a 3–4.5:1 male:female ratio. Patients who underwent Ravitch correction tended to be younger than patients who underwent the Nuss procedure (9.1 *vs* 12.4 years).

Major presenting symptoms included exercise intolerance, dyspnea, and lack of endurance. Western patients were prone to chest pain and cardiopathies, and Asian patients had higher incidences of respiratory tract infection and cardiac displacement.

### FEV1 recovery 1 and 3 years after PE correction

**Figure 1:**
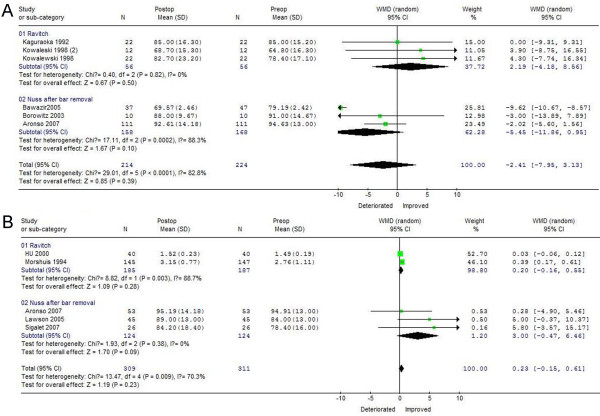
**Forest plot of theRE ORs and 95** **% CIs of theassociation between preoperative FEV1and FEV1 1 yearafter surgical correction (A)and between preoperative FEV1and FEV1 3 yearsafter surgical correction.** (**B**) Surgical correction included the Nuss and Ravitch procedures. The combined estimate is indicated by the diamond. The solid vertical line represents the null result.

As shown in Figure 1A, FEV1 changes after surgical correction favored the Ravitch procedure postoperatively (WMD = 2.19, 95%CI −4.18 ~ 8.56), but favored the Nuss procedure preoperatively (WMD = −5.45, 95%CI −11.86 ~ 0.95). There was evidence of statistically significant heterogeneity among studies (chi-square, 17.11, *p* < 0.05) for the Nuss, but not for the Ravitch, procedure. At 1 year after treatment, FEV1 recovery was better in patients who underwent the Ravitch than the Nuss procedure. However, 3 years after surgical treatment and bar removal, FEV1 changes favored the Nuss procedure (WMD = 3.00, 95%CI −0.47 ~ 6.46), with no significant heterogeneity among studies. For the Ravitch procedure, however, there was evidence of statistically significant heterogeneity among studies (chi-square 8.82, *p* < 0.05; Figure 1B). Thus, FEV1 increased significantly 3 years after surgical correction of PE using a minimally invasive technique, with the Nuss procedure associated with better results than the Ravitch procedure.

### FVC recovery 1 and 3 years after PE correction

**Figure 2:**
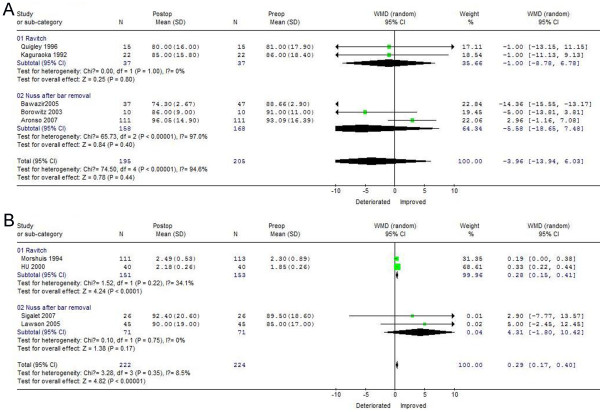
**Forest plot of theRE ORs and 95** **% CIs of theassociation between preoperative FVCand FVC 1 yearafter surgical correction (A)and between preoperative FVCand FVC 3 yearsafter surgical correction (B).** Surgical correction included Nuss and Ravitch procedures. The combined estimate is indicated by the diamond. The solid vertical line represents the null result.

As shown in Figure 2A, FVC changes after surgical correction were good preoperatively for both the Nuss (WMD = −5.58, 95%CI −18.65 ~ 7.48) and Ravitch (WMD = −1.00, 95%CI −8.78 ~ 6.78) procedures. There was evidence of statistically significant heterogeneity among studies for the Nuss (chi-square, 65.73, *p* < 0.05), but not for the Ravitch, procedure. Although FVC decreased within 1 year after surgical correction of PE using both minimal and open techniques, greater FVC improvement occurred 3 years after the Nuss (WMD = 4.31, 95%CI −1.80 ~ 10.42) than after the Ravitch (WMD = 0.28, 95%CI −0.15 ~ 0.41) procedure and bar removal, and there was no evidence of statistically significant heterogeneity for either technique (Figure 2B).

### VC recovery 1 and 3 years after PE correction

**Figure 3:**
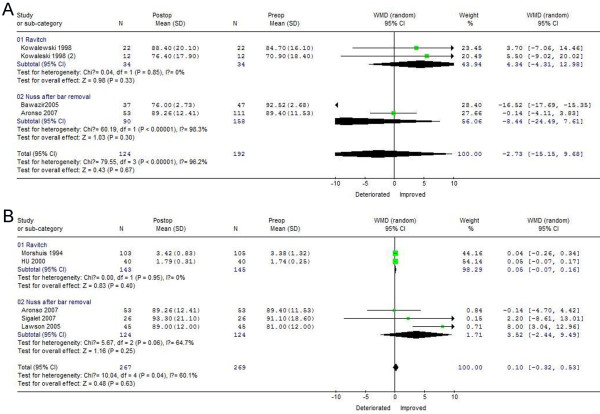
**Forest plot of theRE ORs and 95** **% CIs of theassociation between preoperative VCand VC 1 yearafter surgical correction (A)and between preoperative VCand VC 3 yearsafter surgical correction (B).** Surgical correction included the Nuss and Ravitch procedures. The combined estimate is indicated by the diamond. The solid vertical line represents the null result.

As shown in Figures 3A, 1 year after surgical correction, VC changes favored the Ravitch procedure postoperatively (WMD = 4.34, 95%CI −4.31 ~ 12.98), but favored the Nuss procedure preoperatively (WMD = −8.44, 95%CI −24.49 ~ 7.61), with statistically significant heterogeneity among studies for the Nuss (chi-square, 60.19, *p* < 0.05), but not for the Ravitch, procedure. Three years after surgery and bar removal, however, VC increased significantly, with postoperative results better for the Nuss (WMD = 3.52, 95%CI −2.44 ~ 9.49) than for the Ravitch procedure (WMD = 0.05, 95%CI −0.07 ~ 0.16) procedure, and no evidence of statistically significant heterogeneity among studies for either technique (Figure 3B). These findings indicate that the minimally invasive technique was associated with better improvement in VC after 3 years.

### TLC recovery 1 and 3 years after PE correction

**Figure 4:**
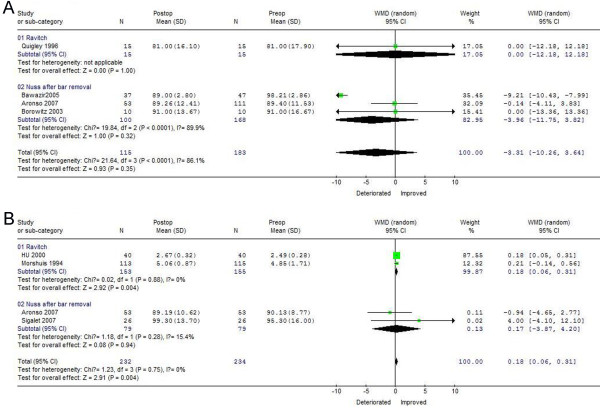
**Forest plot of theRE ORs and 95** **% CIs of theassociation between preoperative TLCand TLC 1 yearafter surgical correction (A)and between preoperative TLCand TLC 3 yearsafter surgical correction (B).** Surgical correction included the Nuss and Ravitch procedures. The combined estimate is indicated by the diamond. The solid vertical line represents the null result.

As shown in Figure 4A, TLC changes after one year favored Nuss correction preoperatively (WMD = −3.96, 95%CI −11.75 ~ 3.82), with statistically significant heterogeneity among studies (chi-square, 19.84; *p* < 0.05). Only one study reported no variation in TLC within 1 year after the Ravitch procedure. Three years after surgery, TLC improved after the Nuss procedure, showed better postoperative results (WMD = 3.52, 95%CI −3.87 ~ 4.20) than the Ravitch procedure (WMD = 0.18, 95%CI 0.06 ~ 0.31), with no evidence of statistically significant heterogeneity among these studies (Figure 4B). These findings indicate that greater increases in TLC after 3 years can be achieved with the Nuss than with the Ravitch technique.

## Discussion

Pectus excavatum is a common chest wall deformity, diagnosed shortly after birth and progressing through adolescence. The defect is often regarded as cosmetic over a long period, primarily because very young patients are often asymptomatic, with symptoms not observed until teenage and later ages [22]. Symptoms in Western patients with PE often include exercise intolerance, dyspnea, chest pain, congenital heart diseases and restrictive pulmonary dysfunction, whereas symptoms in Asian patients include exercise intolerance, dyspnea, frequent or recurrent respiratory tract infections and restrictive pulmonary dysfunctions. Cardiac function improvement after correction of PE has already been demonstrated in individual studies and meta-analyses, and our results, showing that cardiovascular function after surgery improved by greater than one-half standard deviation, support the hypothesis that relief of cardiac compression caused by the depressed sternum improves the hemodynamic responses of patients with PE [27].

By contrast, pulmonary function improvement after surgical treatment of PE has not yet gained widespread acceptance. Few studies have compared surgically treated and untreated patients with PE, and many studies have used patients as their own controls. We therefore analyzed whether pulmonary functional recovery occurred after the surgical correction of PE, and whether this recovery differed by type of procedure performed or by time after surgery.

Although PE patients with mild sternal depression seem unlikely to benefit greatly from surgical correction, partly because the intrathoracic volume might not be significantly reduced [3], it is unclear whether the pathophysiologic deficits in PE are primarily ventilatory or cardiovascular (or both). These deficits are caused by the displaced sternum compressing the right ventricular outflow tract, which may cause dyspnea, recurrent respiratory tract infection, and pulmonary insufficiency, all indicators of diminished lung capacity. Thus, surgical management may improve a patient's exercise tolerance and relieve symptoms such as poor lung capacity [28,29]. This is particularly applicable to patients with severe deformities, because surgery may reduce intrathoracic volume considerably, making space available for smooth lung expansion. More importantly, we identified several studies that showed a modest preoperative reduction in vital capacity and total lung capacity, both of which improved after surgical correction [5].

We also assessed whether pulmonary functional recovery was dependent on type of surgical procedure. We found that forced expiratory volume, vital capacity, and total lung capacity did not increase greatly after either the Nuss or Ravitch procedure but before bar removal, whereas significant improvements in lung function were observed after bar removal. However, better long-term ventilatory outcomes were observed after Nuss correction. Specially, we observed slight differences in pre- and post-operative pulmonary function in patients with mild-to-moderate PE [38]. In contrast, higher pulmonary function capacity after surgery may have been due to a restriction secondary to the Nuss operation after removal of the bar. In patients with an abnormal chest wall, the effect of the extrinsic musculature may be compromised, and diaphragmatic efficiency is likely reduced [39]. Conceptually, correction of the chest wall abnormality should improve both intrinsic musculature and diaphragmatic function. That this occurs is evidenced by the selective improvement of the most dynamic component of pulmonary function, FEV1.

We observed recurrence of sternal depression after surgical correction during the pubertal growth spurt, suggesting that costal cartilage resection may be unnecessary during surgical repair of prepubertal patients. Unfortunately, we were unable to find studies with similar findings for a meta-analysis. Whereas the extraction force in adolescents is about 175 N, the average force in adults was greater than 200 N, making it impossible to elevate the sternum to the desired level in adults without surgical mobilization. Only about 50% of this tension can be eliminated by costal chondrotomy. This may contribute to the recurrence of PE after correction, suggesting a need for costal cartilage resection [40,41].

Patient age at the time of surgery is important for both speed of recovery and long-term results [42]. Before puberty children have a soft and malleable chest and therefore recover from surgical repair and return to regular activity earlier than do adolescents and adults. Long-term results, however, are better in older patients, especially if they have completed their growth at the time of bar removal. Since regression or improvement of PE is rarely due to the fixation of cartilage and ligaments, the ideal age for optimal PE correction should be around puberty, when the costal cartilages are still flexible and malleable. This coincides with the growth spurt and can allow a better observation of the outcome of repair and timely intervention in patients who need revision surgery [43].

One limitation of our data was our inclusion of only surgical candidates, who are by definition more likely to have at least moderately restricted chest wall function. Thus, our results may not be generalizable to all patients with PE. Our findings were also limited by the absence of uniformity in recording pulmonary function tests, suggesting the need for objective pulmonary function tests that would be less affected by patient subjectivity.

## Conclusions

Although the Ravitch and Nuss procedures for the correction of PE tended to show the same improvements in pulmonary function within 1 year, the Nuss procedure was associated with greater improvements in pulmonary function after bar removal. There is a need for further large-scale prospective trials and meta-analyses, based on the homogeneity of assessment parameters, to determine the principles of PE regression or recurrence over time, and better assess the extent of the beneficial effects of PE correction on pulmonary function.

## Abbreviations

CI: Confidence interval; FEV1: Forced expiratory volume over 1 second; FVC: Forced vital capacity; PE: Pectus excavatum; NA: Nonapplicable; NR: Non reported; Postop: Postoperative; Preop: Preoperative; PSI: Pectus severity index; VC: Vital capacity; TLC: Total lung capacity; WMD: Weighted mean difference.

## Competing interests

Drs. Zhenguang Chen, Honghe Luo, Amos Ela Bella, Chunhua Su, Beilong Zhong, Jianyong Zou, and Yiyan Lei have no competing interest or financial ties to disclose.

## Authors' contributions

ZC and EBA conceived the study, performed papers collection and statistical analysis of the paper. HL participated in the manuscript preparation and coordinating of the study. CS and BZ participated in the statistical analysis of all data. JZ and YL participated in the drafting of the manuscript. All authors have read and approved the final version of the manuscript.
